# An Unusual Case of Acute Cholecystitis Caused by Lactobacillus paracasei

**DOI:** 10.7759/cureus.40334

**Published:** 2023-06-12

**Authors:** Hui Un Kim, Byeongyeon Choo, Arun Pyakuryal, Munir Shah

**Affiliations:** 1 Internal Medicine, American University of Antigua, New York City, USA; 2 Internal Medicine, Western Reserve Health Education/Northeast Ohio Medical University (NEOMED), Warren, USA; 3 Infectious Disease, Western Reserve Health Education/Northeast Ohio Medical University (NEOMED), Warren, USA

**Keywords:** bile culture, gallbladder, percutaneous cholecystostomy, lactobacillus, acute cholecystitis

## Abstract

*Lactobacillus* *paracasei* is a gram-positive rod commonly found in probiotic foods and is well known to promote healthy gastrointestinal tracts. However, there have been a few case reports that have found *Lactobacillus paracasei* to be the causative agent in complications such as endocarditis, meningitis, peritonitis, pancreatitis, and cholecystitis.

We present the case of a 76-year-old woman who was admitted for decompensated heart failure. The patient also reported abdominal pain in the right upper quadrant. Ultrasound findings suggested cholelithiasis and a subsequent hepatobiliary iminodiacetic acid (HIDA) scan confirmed acute cholecystitis. This patient was not a good candidate for cholecystectomy because of the risk of cardiac complications. Hence, percutaneous cholecystostomy tube (PCT) placement was done, and the aspirate drained during the procedure was sent for pathology. This bile culture was positive for *Lactobacillus paracasei* and negative for any other kinds of bacteria.

The incidence of *Lactobacillus* species-induced cholecystitis makes up only 0.08% to 0.2% of cases, which makes this an unusual case of acute cholecystitis caused by *Lactobacillus paracasei*. We will discuss several pathogenic aspects of *Lactobacillus paracasei, *such as its ability to generate biofilms, pore-forming toxins, drug transporters, and antibiotic susceptibility.

## Introduction

*Lactobacillus *species are gram-positive rods that produce lactic acid, which is commonly found in the flora of the oral, gastrointestinal, and female genital tracts [1]. *Lactobacillus paracasei* is widely used in dairy food fermentation and probiotic cultures and is known to promote healthy gastrointestinal tracts by improving immune function and preventing pathogenic microbes from colonizing the gastrointestinal tract [2,3]. However, studies have also shown that *Lactobacillus *species can also be the causative agent for bacterial endocarditis, meningitis, peritonitis, pancreatitis, and possibly cholecystitis [4-10]. In this case, *Lactobacillus paracasei* was the main pathogen that induced acute cholecystitis.

## Case presentation

**Figure 1:**
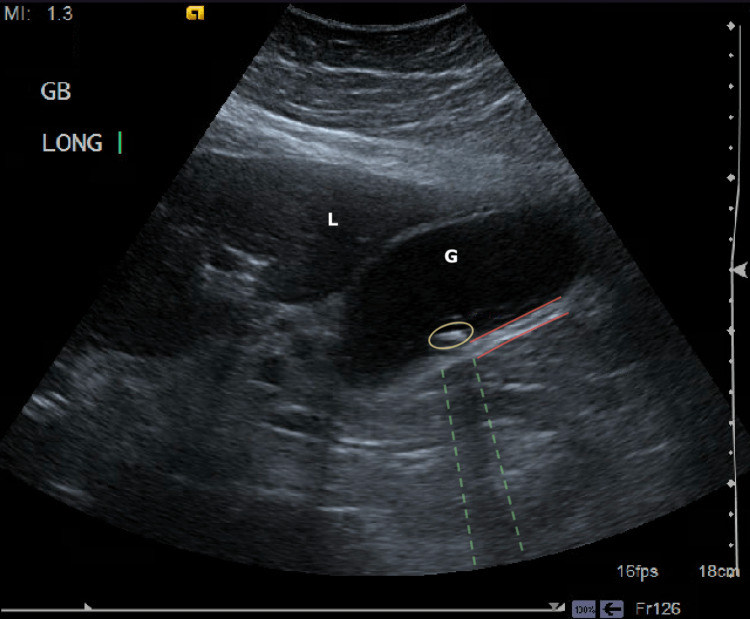
Ultrasound of the gallbladder longitudinal plane with the findings of cholelithiasis and acute cholecystitis Hyperechoic calculi are marked in the yellow circle, with acoustic shadowing seen in the dashed green lines. The red lines outline the thickened gallbladder wall at 4.8 millimeters (mm) (G: gallbladder; L: liver).

**Figure 2:**
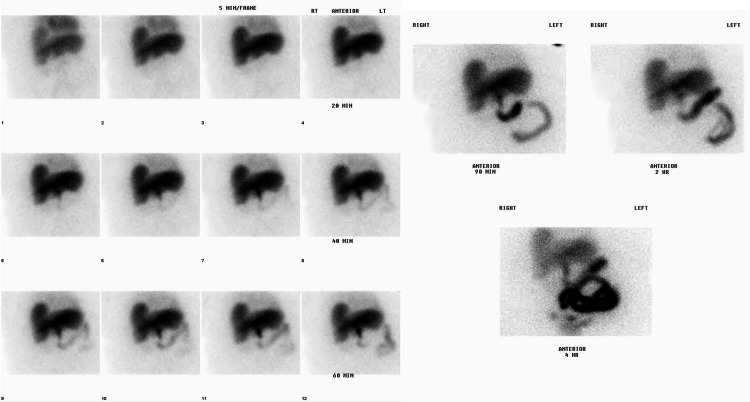
HIDA scan indicating acute cholecystitis There is no visualization of the gallbladder in this image, which confirms obstruction of the cystic duct, indicating acute cholecystitis.

A 76-year-old woman with a medical history of atrial fibrillation, congestive heart failure, appendectomy, type II diabetes, and hypertension was admitted for acute and chronic systolic heart failure with New York Heart Association (NYHA) class III-IV. On physical exam, the patient had right upper quadrant pain with mild tenderness but a negative Murphy sign. The lab result showed leukocytosis with white blood cells (WBC) trending up from 17.9 to 23.4 thousand cells per microliter (10^3^ cells/μL), partial thromboplastin time of 33.6 seconds (s), the international normalized ratio of 3.2, blood glucose of 242 milligrams per deciliter (mg/dL), up trending total bilirubin of 0.8 to 4.8 mg/dL, up trending liver function test with aspartate transaminase (AST) of 296 units per liter (U/L) and alanine transaminase (ALT) of 190 U/L, and alkaline phosphatase (ALP) of 592 U/L (Table 1). Findings of the ultrasound of the right upper quadrant suggested cholelithiasis and possible cholecystitis with gallbladder wall thickening, and a hepatobiliary iminodiacetic acid (HIDA) scan was done subsequently to confirm acute cholecystitis (Figures 1-2).

**Table 1:** Laboratory results pertinent to acute cholecystitis WBC: white blood cells; AST: aspartate transaminase; ALT: alanine transaminase; ALP: alkaline phosphatase

	On admission	Day two	Reference values
WBC (10^3^cells/μL)	17.9	23.4	3.5 - 10.5
Total bilirubin (mg/dL)	0.8	4.8	0.2 - 1.0
Direct bilirubin (mg/dL)	-	3.2	0.0 - 0.2
AST (U/L)	51	296	10 - 37
ALT (U/L)	55	190	12 - 78
ALP (U/L)	313	592	45 - 117

General surgery was consulted on this case for possible cholecystectomy, but the patient was not deemed a surgical candidate due to acute or chronic systolic heart failure with NYHA class III-IV. The plan was to assess the patient for possible surgical intervention and monitor her in conjunction with the cardiologist on board with this case. During this time, the patient had been receiving metronidazole and ceftriaxone for six days. On day seven of the admission, there was no improvement in the heart condition; hence, interventional radiology was consulted for a percutaneous cholecystostomy. Computed tomography (CT)-guided percutaneous drainage of the gallbladder was done with the anterior approach passing through the left hepatic lobe. Approximately 8 milliliters (mL) of dark green/brown purulent fluid were aspirated and sent to pathology for further evaluation.

The culture report of the bile acid presented an atypical finding. The cultures were positive for gram-positive rods, identified as *Lactobacillus paracasei *using Vitek®. Otherwise, the cultures were negative for any other bacteria. For this reason, infectious disease was consulted for the management of this atypical case of acute cholecystitis. Metronidazole and ceftriaxone were discontinued, and ampicillin and sulbactam were initiated to cover *Lactobacillus paracasei*. Unfortunately, antibiotic susceptibility was not obtained because there was a miscommunication with the lab and the sample was no longer available by the time the test was ordered again.

The patient was admitted for another six days after the percutaneous cholecystostomy tube placement for medical management of her congestive heart failure and cholecystitis. During this time, there was minimal drainage of the bile acid from the cholecystostomy tube, and another sample could not be obtained. Then she was discharged on amoxicillin and clavulanic acid for another 10 days as she was medically stable and was advised to follow up with the surgeon who was on board with this case 10 days after discharge.

Twenty days after discharge, the patient was readmitted to the hospital for acute hypoxic respiratory failure and congestive heart failure exacerbations. There was no follow-up on the cholecystostomy tube with the surgeon prior to this second admission. The CT scan of the abdomen on this admission showed multiple gallstones but no signs of acute cholecystitis or significant bile duct dilation. The surgeon decided to remove the dysfunctional cholecystostomy tube and leave it out, and no surgical intervention was done as the patient was not deemed a surgical candidate due to a heart failure exacerbation.

## Discussion

*Lactobacillus paracasei*, well known to be beneficial for gastrointestinal health and also known to aid in alleviating symptoms of irritable bowel syndrome, is also known to be pathogenic, potentially causing a few complications [2,3,8,9]. There have been only a few case reports of *Lactobacillus paracasei* being the major causative bacteria in conditions such as bacterial endocarditis, meningitis, peritonitis, and cholecystitis, as seen in this case [5,8-10].

The most common etiologies of biliary tract infection are *Escherichia coli* (31-44%), *Klebsiella spp.* (9%-20%), *Enterococcus spp.* (3%-34%), and *Enterobacter spp.* (5%-9%) [4]. The chances of finding *Lactobacillus *in the gallbladder are very low, and the incidence of *Lactobacillus* species-induced cholecystitis makes up only 0.08% to 0.2% of cases [5,6]. Further, a retrospective study showed only a few reports of *Lactobacillus *as the primary pathogen in the setting of cholecystitis from 200 Lactobacillus-related reports [7]. Antibiotic treatment with metronidazole and ceftriaxone had already been initiated prior to the bile acid collection, and there is a possibility the antibiotics may have affected the culture result. However, the culture of the bile acid showed many WBCs, many gram-positive rods on gram stain, and a light growth of *Lactobacillus paracasei* in spite of antibiotic treatment with metronidazole and ceftriaxone, implicating an atypical case of cholecystitis.

Considering all the risk factors this patient had, the pathogenicity of *Lactobacillus spp.* should be mentioned. First, *Lactobacillus paracasei *is able to produce biofilm, which may have contributed to colonization in the gallbladder and increased antibiotic resistance compared to other bacterial species frequently found in acute cholecystitis [8,11,12]. This may have contributed to colonization and increased antibiotic resistance compared to the other bacterial species in the gallbladder, as seen in the culture report. In addition, Rossia et al. propose that *Lactobacillus paracasei*, along with the other *Lactobacillus *species (*L. plantarum *and *L. rhamnosus*), have a higher pathogenic potential that can make pore-forming toxins and drug exporters [11]. These species also have a much higher number of other membrane transporters, such as amino acid transporters, sugar transporters, and other unidentified transporters, allowing them to survive better than other species and cause infections [12].

We believe that this *Lactobacillus *species isolated in the bile was a pathogen as opposed to mere colonization since it was isolated from an unusual site of growth. We suspect migration of the *Lactobacillus *species in the gastrointestinal tract due to alteration of the colonic microbiota. The factors that commonly affect the composition of the gastrointestinal microbiota are the use of antibiotics, age, and diet [11]. Also, bacterial transmigration is more likely to occur in individuals with diabetes [12]. Diabetics with hyperglycemia, as seen in this patient, are more prone to *Lactobacillus paracasei* infection, which increases the availability of glucose for bacterial metabolism and thereby aids in adherence and biofilm formation as a protective measure [8,11,12]. Also, diabetic patients have increased vascular permeability, which provides *Lactobacillus paracasei *with a better environment to transmigrate into other systems of the body than in non-diabetics and cause infections in unusual locations, as seen in this patient with acute cholecystitis [12].

The use of probiotics is beneficial if they are consumed in adequate amounts, but we also believe that the increased intake of yogurt seen in this patient may have possibly contributed to this case as it is a common source of *Lactobacillus paracasei *[12,13]. Costa et al. report *Lactobacillus*-induced bacteremia as the second most common complication from probiotic use, which accounts for 27.9% of the cases in their study [13]. The use of probiotics needs to be carefully assessed, especially in individuals of age 60 and above, the immunocompromised, and diabetic individuals [12,13]. In addition to bacteremia, acute cholecystitis is also a possible complication that needs to be addressed. A feared complication of acute cholecystitis is the progression to secondary bacterial peritonitis, which can further progress to bacteremia, which can lead to fatal complications such as endocarditis and meningitis [5-10].

The antibiotic susceptibility pattern is important in the management of *Lactobacillus *species-related complications. *Lactobacillus spp.* are reported to be susceptible mainly to beta-lactams [5,8,10,12,14].​​​​ Other susceptible antibiotics reported are chloramphenicol, tetracycline, erythromycin, linezolid, and quinupristin-dalfopristin [14]. In addition, *Lactobacillus* species are found to be resistant to vancomycin, aminoglycosides, ciprofloxacin, and trimethoprim [14]. *Lactobacillus paracasei* found in this case suggests possible antibiotic resistance to ceftriaxone and metronidazole, as it was found in the bile acid culture even after six days of administration of these two antibiotics. There is another case report of acute cholecystitis caused by *Lactobacillus paracasei*, which was resistant to cefotaxime and metronidazole, that supports our concerns for possible species- or strain-related antibiotic resistance to cephalosporins and metronidazole in *Lactobacillus paracasei *[5]. This case report, along with the other case reports, serves as a good example of *Lactobacillus paracasei* being sensitive to beta-lactams such as ampicillin/sulbactam, amoxicillin/clavulanic, piperacillin/tazobactam, and imipenem [5,8,10,12]. Our findings suggest antibiotic resistance to cephalosporins as a possibility in *Lactobacillus paracasei*, in spite of it being susceptible to other beta-lactams mentioned above.

Antibiotic resistance to cephalosporins in *Lactobacillus paracasei* was also brought to our attention, and the proposed mechanism is assumed to be the impermeability of the cell wall [8]. Vancomycin is reported to be ineffective in the management of *Lactobacillus* species-related complications [5,8,10,12,14]. The suggested mechanism of vancomycin resistance is the alteration of the vancomycin binding site, where dextro (D) alanine residue is replaced with D-lactate or D-serine [14]. Metronidazole is normally used to cover community-acquired biliary infections for anaerobic coverage [4]. However,* Lactobacillus paracasei *is a facultative anaerobic or microaerophilic bacteria, which explains its resistance to metronidazole, consistent with our finding [5,8].

## Conclusions

This is an unusual case of acute cholecystitis caused by *Lactobacillus paracasei *as the primary pathogen. Although studies have shown many benefits from this bacteria, it could also be pathogenic on rare occasions. We speculate that increased intake of probiotics in individuals of age 60 and above and diabetic individuals may have possibly increased the risk for infectious complications. We propose a few pathogenic mechanisms of *Lactobacillus paracasei*, such as its utilization of biofilms, pore-forming toxins, and drug exporters, that may have promoted colonization in the gallbladder, causing acute cholecystitis. Antibiotic management in our case suggests antibiotic susceptibility to beta-lactams in *Lactobacillus paracasei*, consistent with the findings of the other case reports. In addition, the findings of this study raise concern for possible antibiotic resistance to cephalosporins in *Lactobacillus paracasei*.

More studies on *Lactobacillus spp*. and their pathogenic potential can help us understand this condition better and prevent complications. Until now, there have not been many studies on *Lactobacillus spp*. acting as the primary pathogen in acute cholecystitis, as seen in this case, and other complications such as endocarditis, meningitis, peritonitis, and pancreatitis. More studies on the pathogenicity of *Lactobacillus *spp. can help develop preventive measures and better antibiotic coverage that would cover pathogenic *Lactobacillus spp*. to prevent the unforeseen complications mentioned above.
